# *Salmonella* Typhimurium induces SPI-1 and SPI-2 regulated and strain dependent downregulation of MHC II expression on porcine alveolar macrophages

**DOI:** 10.1186/1297-9716-43-52

**Published:** 2012-06-13

**Authors:** Alexander Van Parys, Filip Boyen, Elin Verbrugghe, Bregje Leyman, Flahou Bram, Freddy Haesebrouck, Frank Pasmans

**Affiliations:** 1 Ghent University, Faculty of Veterinary Medicine, Department of Pathology, Bacteriology and Avian Diseases, Salisburylaan 133, 9820, Merelbeke, Belgium

## Abstract

Foodborne salmonellosis is one of the most important bacterial zoonotic diseases worldwide. *Salmonella* Typhimurium is the serovar most frequently isolated from persistently infected slaughter pigs in Europe. Circumvention of the host’s immune system by *Salmonella* might contribute to persistent infection of pigs. In the present study, we found that *Salmonella* Typhimurium strain 112910a specifically downregulated MHC II, but not MHC I, expression on porcine alveolar macrophages in a *Salmonella* pathogenicity island (SPI)-1 and SPI-2 dependent way. *Salmonella* induced downregulation of MHC II expression and intracellular proliferation of *Salmonella* in macrophages were significantly impaired after opsonization with *Salmonella* specific antibodies prior to inoculation. Furthermore, the capacity to downregulate MHC II expression on macrophages differed significantly among *Salmonella* strains, independently of strain specific differences in invasion capacity, *Salmonella* induced cytotoxicity and altered macrophage activation status. The fact that strain specific differences in MHC II downregulation did not correlate with the extent of *in vitro* SPI-1 or SPI-2 gene expression indicates that other factors are involved in MHC II downregulation as well. Since *Salmonella* strain dependent interference with the pig’s immune response through downregulation of MHC II expression might indicate that certain *Salmonella* strains are more likely to escape serological detection, our findings are of major interest for *Salmonella* monitoring programs primarily based on serology.

## Introduction

Nontyphoidal salmonellosis is one of the most important bacterial zoonotic diseases, yearly resulting in an estimated 155 000 deaths worldwide [1]. In European countries, *Salmonella enterica* subspecies *enterica* serovar Typhimurium (*Salmonella* Typhimurium) is the serovar most frequently isolated from slaughter pigs [2]. Pig carcass contamination with *Salmonella* Typhimurium can largely be attributed to persistently infected pigs [3]. In most cases, the bacterium will asymptomatically colonize pigs, resulting in a so called “carrier status” [4,5]. In the past, *Salmonella* infections in pig herds have traditionally been diagnosed by culturing intestinal or faecal samples [6]. Because pigs only excrete high numbers of bacteria during the acute phase of infection and then become intermittently excreting carriers, serological surveillance is perceived as a practical (high-throughput) and cost-effective alternative for monitoring *Salmonella* infection in pig herds [6-9].

After infection by a pathogen, the host’s immune system will respond through an innate and a subsequent adaptive immune response. The success of many persisting pathogens relies on their ability to resist, circumvent or counteract the host’s innate and/or adaptive immune responses. Several bacteria and viruses have developed pathways interfering with antigen presentation by the host’s immune system, for example by inhibition of MHC expression and antigen presentation through distinct mechanisms [10-12]. The *Salmonella* genome contains several *Salmonella* pathogenicity islands (SPIs’), clusters of genes that encode virulence factors involved in different stages of *Salmonella* pathogenicity [13]. SPI-2, encoding a type III secretion system, plays a role in *Salmonella*-mediated downregulation of cell surface MHC class II expression in human cell lines by recruiting membrane for the *Salmonella* containing vacuole [12]. However, *Salmonella* exhibits host specific behaviour and no data are available yet on this phenomenon in pigs. In addition, it is not known whether different *Salmonella* strains might be able to interfere differentially with the MHC II expression pathway in porcine cells. By manipulating the porcine humoral immune response, certain *Salmonella* strains might be able to persist better than strains that do not interfere with the immune response in pigs.

Besides direct SPI mediated effects on MHC recruitment, expression of MHC molecules may be affected by other *Salmonella* induced effects on macrophages, like cytotoxicity and altered macrophage activation status. *Salmonella* infection of porcine macrophages results in a type of cell death called pyroptosis. In contrast to apoptosis, *Salmonella* induced pyroptosis affects plasma membrane integrity and might thus interfere with the expression of macrophage surface molecules [14]. Furthermore, *Salmonella* infection of mammalian cells induces the production of reactive oxygen species (ROS) as part of the cellular immune response to eradicate intracellular pathogens and might also interfere with the expression of surface molecules [15,16].

In the present study, we examined whether a *Salmonella* Typhimurium strain, which can persist in pigs, is able to downregulate MHC expression on porcine macrophages in a SPI-1 and/or SPI-2 dependent way, as a possible mechanism to circumvent antibody production by the pig’s immune system. Furthermore, in an attempt to elucidate the importance for *Salmonella* to interfere with the antibody response in pigs, we verified the role of *Salmonella* specific antibodies in the bacterium’s ability to interfere with the MHC presentation pathway and in intracellular proliferation of *Salmonella* in porcine macrophages. Finally, we examined whether different *Salmonella* strains exhibit similar effects on MHC II expression.

## Materials and methods

### Bacterial strains and manipulations

*Salmonella* Typhimurium strain 112910a was isolated from a pig stool sample and several experimental infections showed that this strain was able to persistently infect piglets [17,18] without inducing significant seroconversion until at least 4 weeks post inoculation [unpublished observations]. *Salmonella* Typhimurium strain 112910a *hilA* and *ssrA/B* deletion mutants (hereafter named Δ*hilA* and Δ*ssrA/B*, respectively) were constructed according to the one-step inactivation method described by Datsenko and Wanner [19] and slightly modified for use in *Salmonella* Typhimurium as described before [20,21]. Subsequently, the kanamycin resistance cassette was removed using the helper plasmid pCP20 [19]. The *hilA* gene and the *ssrA/B* operon encode major SPI-1 and SPI-2 regulators, respectively [22,23]. Gene complementations for the deletion mutants Δ*hilA* and Δ*ssrA/B* were constructed using vector plasmid pGV1106 [24]. In short, plasmid pGV1106 was digested with *Eco*RI and *Bgl*II restriction enzymes (New England Biolabs, Ipswich, Massachusetts, USA), ligated with the PCR amplified linear *hilA* gene or *ssrA/B* operon (primers are given in Table 1) and electroporated into electrocompetent *Escherichia coli* K514 and plated on Luria-Bertani agar (LB agar; Sigma-Aldrich, Chemie Gmbh, Steinheim, Germany) with 25 μg/mL kanamycin (Sigma-Aldrich). The resulting complementation plasmids were isolated and *hilA* and *ssrA/B* inserts were sequenced to verify if the promoter sequence and the complete coding sequence were inserted. pGV1106 plasmids containing the *hilA* gene or *ssrA/B* operon insert were then electroporated in the respective deletion mutants. The resulting complemented deletion mutants are further abbreviated as Δ*hilA*^c^ or Δ*ssrA/B*^c^, respectively.

**Table 1:** Primers used in this study

Name	Sequence
HilAcomplFW	5′-gggagatctgtaaaacgacggccagtcggctttaaccctgtggatt-3′
HilAcomplRV	5′-ggggaattcggataacaatttcacacaggtgcatatctcctctctcagat-3′
SsrA/BcomplFW	5′-gggagatctgaatgcattagacatgcctcggtcagtcgtttagcct-3′
SsrA/BcomplRV	5′-ggggaattcacatagctatttttctgatcggggctaaatgctagtcct-3′

Primers used to create the Δ*hilA* and Δ*ssrA/B* gene complementations.

In addition to *Salmonella* Typhimurium strain 112910a, other strains used in this study were *Salmonella* Typhimurium pig isolates MB2150, MB2216, MB2222, MB2223, MB2233 and MB2498 and the pigeon isolate DAB69 [25], one isolate of *Salmonella* serovars Brandenburg, Derby and Infantis, all isolated from pigs and a chicken isolate *Salmonella* serovar Enteritidis 76Sa88 [26]. Finally, an *Escherichia coli* strain p4:o32 isolated from a bovine mastitis case was tested [27].

For flowcytometrical analysis of *Salmonella* infected macrophages, all *Salmonella* strains were transformed with the pFPV25.1 carbenicillin-resistant plasmid expressing green fluorescent protein (GFP) under the constitutive promoter of *rpsM*[28]. For inoculation of macrophages, bacteria were grown for 16 h at 37 °C in 5 mL LB broth with 25 μg/mL carbenicillin (Sigma-Aldrich) for selective growth of GFP-transformed bacteria.

### Pig antisera against *Salmonella* Typhimurium

Pig antisera were raised in *Salmonella*-negative piglets, as approved by the ethical committee of the Faculty of Veterinary Medicine, Ghent University (EC2008/124). To prepare positive serum, piglets were intramuscularly injected twice, with a 3 weeks interval, with 2 mL of a bacterin consisting of equal volumes of formalin-inactivated *Salmonella* Typhimurium strain 112910a suspended in phosphate buffered saline (PBS; 5 × 10^8^ colony forming units (CFU) per mL, before inactivation) and Freund’s incomplete adjuvant. Negative serum was prepared in piglets injected twice with 2 mL of an emulsion consisting of equal volumes of PBS and Freund’s incomplete adjuvant. Blood samples were collected from the jugular vein using VenoJect blood collection tubes (Terumo Europe N.V., Leuven, Belgium). The blood samples were incubated for 1 h at 37 °C to facilitate blood clotting and were then centrifuged for 10 min at 520 × *g* and 20 °C. Serum was collected and tested for the presence of *Salmonella* specific antibodies using a commercially available LPS-based swine *Salmonella* Typhimurium test kit (IDEXX Europe B.V., Hoofddorp, The Netherlands) according to the manufacturer’s guidelines. Serum samples were stored at -80 °C until further use.

Bacteria were opsonized by adding an equal volume of pig serum to a *Salmonella* Typhimurium 16 h culture, containing approximately 1 × 10^9^ CFU per mL, and by subsequent incubation for 20 min at 37 °C on a shaker. For complement inactivation, the sera were placed at 56 °C for 10 min prior to opsonization.

### Macrophage assays

Porcine alveolar macrophages were isolated from lungs of humanely euthanized *Salmonella*-free piglets as described previously [29]. Using this isolation method, the purity of the macrophage pellets varied between 90% and 97% and the viability was more than 95% [20]. Macrophage pellets were suspended in foetal calf’s serum (FCS; Gibco Life Technologies, Ghent, Belgium) with 10% DMSO (Sigma-Aldrich) and stored in liquid nitrogen at approximately 10^7^ cells per mL until further use. Prior to seeding, macrophages were thawed at room temperature, washed in 10 mL Hank’s buffered salt solution (HBSS; Gibco) containing 10% FCS (and without FCS for the macrophage activation assay) and centrifuged at 520 × *g* for 10 min at 4 °C. The pellet was resuspended in RPMI medium composed of Roswell Park Memorial Institute medium with phenol red (RPMI; Gibco), 10% v/v FCS, 2 mM sodium pyruvate (Gibco), 2 mM L-glutamine (Gibco), 1 mL v/v MEM non essential amino acids 100× (Gibco) and 100 μg/mL penicillin and streptomycin (Gibco), unless otherwise stated. The number of macrophages per mL as well as their viability was determined using trypan blue exclusion and a counting chamber (Bürker, Marienfeld, Germany). All macrophage assays were conducted 3 times independently in triplicate, unless otherwise stated, with macrophages from 3 different pigs.

#### The effect of *Salmonella enterica* infection on MHC expression on porcine macrophages

Macrophages were seeded in a 29-well plate in 25 cm² tissue culture treated flasks with filter cap (Greiner Bio-One) at 5 × 10^6^ cells per flask. Macrophages were allowed to adhere for 2 h at 37 °C and were then inoculated with *Salmonella* Typhimurium strain 112910a that was either not opsonized, opsonized with porcine serum containing anti-*Salmonella* Typhimurium antibodies or opsonized with negative pig serum, at a multiplicity of infection (MOI) of 10. Flasks were centrifuged at 520 × *g* for 10 min at 37 °C and subsequently incubated at 37 °C for 30 min. Macrophages were washed 3 times with HBSS containing calcium and magnesium (HBSS+; Gibco) and treated with 100 μg/mL gentamicin (Sigma-Aldrich) in RPMI for 1 h at 37 °C to kill all extracellular bacteria. The cells were washed again 3 times with HBSS+ and fresh medium was added. This timepoint was considered as 0 h post inoculation. Macrophages were scraped off of the flask’s bottom at 0 and 24 h post inoculation using rubber cell scrapers (Greiner Bio-One), washed with 5 mL ice-cold HBSS and resuspended in medium. These suspensions were equally distributed over 2 flowcytometry tubes (Becton-Dickinson Labware Europe, Meylan-Cedex, France). Macrophages were incubated with 10% complement inactivated *Salmonella*-negative goat serum and 10% complement inactivated *Salmonella*-negative pig serum for 15 min on ice to reduce background staining. After centrifugation, macrophage pellets were resuspended in a primary antibody mixture or in RPMI for the negative controls. The primary antibody mixtures were composed of RPMI, 15% FCS and 2% mouse anti-pig SLA class I (1:50 diluted; AbD Serotec, Kidlington, Oxford, UK) or 20% mouse anti-pig SLA class II DQ (1:5 diluted; AbD Serotec). After 50 min incubation on ice, cells were washed with ice-cold HBSS to remove unbound antibodies. After centrifugation, macrophage pellets were resuspended in a secondary antibody mixture or RPMI for the negative control tubes, and incubated for 50 min on ice and in the dark. The secondary antibody mixture contained RPMI, 15% FCS and 2% goat anti-mouse Alexa Fluor 633 antibody (1:50 diluted; Molecular Probes, Oregon, USA). Macrophages treated with only the primary or secondary antibody mixture served as controls. After a final washing step, the pellets were resuspended in 0.5 mL ice-cold HBSS for flowcytometric analysis. Flowcytometric measurements were performed using a FACScanto^TM^ II cytometer (Becton–Dickinson, Erembodegem, Belgium). First, macrophages were discriminated from bacteria and debris based on forward (FSC) and side (SSC) light scatter (parent population). Subsequently, *Salmonella* infected macrophages were distinguished from uninfected macrophages in the parent population, based on GFP fluorescence using the FL-1 fluorescence channel, and the MHC I and II expression levels of infected and uninfected macrophages were measured using the FL-5 fluorescence channel. Data were expressed in arbitrary units and the mean FL-5 value of *Salmonella* infected and uninfected macrophages were calculated using the FACSDiva software (Becton–Dickinson).

Exactly the same procedure was used for macrophage infection and subsequent detection of MHC II expression levels with all other strains and mutants used in this study.

#### Invasion capacity

As a measure for the *Salmonella* invasion capacity in macrophages, the number of macrophages infected by strain 112910 that was opsonized with negative or positive pig serum, or by any other *Salmonella* strain at 0 h pi, as assessed by measurement in the FL-1 fluorescence channel, was expressed as the percentage relative to the macrophages infected by strain 112910a at the same timepoint. *Salmonella* Typhimurium Δ*hilA*, that is impaired in macrophage invasion [20], and its complement Δ*hilA*^c^ were used as internal controls for the use of flowcytometrical data to assess *Salmonella* invasion capacity.

#### Macrophage viability assay

We used the neutral red uptake assay to assess possible cytotoxic effects of *Salmonella* infection on macrophage viability. Macrophages were cultured and inoculated in a 96-well plate as described above for the flowcytometry experiments. Twenty-four hours after inoculation, 200 μL of freshly prepared neutral red solution (33 μg/mL neutral red (Merck N.V./S.A., Overijse, Belgium) in RPMI without phenol red), prewarmed at 37 °C, was added to each well and plates were incubated for 2 h at 37 °C. Macrophages were washed 3 times with HBSS+ and treated with 170 μL extracting solution, composed of absolute ethanol/distilled water/glacial acetic acid 50/49/1 (v/v/v), for 10 min at room temperature on a shaker to release the neutral red dye. Hundred microliter from each well were transferred to an IWAKI multi well plate and the absorbance was determined at 540 nm using a microplate ELISA reader (Multiscan MS, Thermo Labsystems, Helsinki, Finland). Macrophage cell viability at 24 h post inoculation (pi) was expressed as the percentage of viable macrophages compared to uninfected macrophages at this timepoint.

#### Impact of opsonization with antibodies on intracellular proliferation of *Salmonella* Typhimurium in porcine macrophages

Macrophages were seeded in a 29-well plate at 2 × 10^5^ per well and were inoculated at MOI 10 with *Salmonella* Typhimurium strain 112910a that was either non-opsonized, opsonized with porcine serum containing *Salmonella* Typhimurium specific antibodies or opsonized with negative pig serum. Macrophages were then treated with 100 μg/mL gentamicin as described above. After 0 or 5 h incubation at 37 °C, macrophages were lysed using 0.2% Triton X-100 (Sigma-Aldrich) and 10-fold dilutions were plated on brilliant green agar (BGA; International Medical Products N.V./S.A., Brussels, Belgium) to enumerate viable intracellular bacteria.

#### Macrophage activation assay

To assess the influence of *Salmonella* infection on the macrophage activation status, the production of reactive oxygen species (ROS) by macrophages was measured using the lucigenin assay [30]. Macrophages were suspended in RPMI medium without phenol red and without FCS, and seeded at 2 × 10^5^ cells per well in a 96-well cell culture plate (Greiner Bio-One). After 2 h incubation at 37 °C, lucigenin (Sigma-Aldrich) solution at a final concentration of 400 μM and *Salmonella* were added to each well at MOI 10. Positive control wells were treated with 20 μg/mL phorbol 12-myristate 13-acetate (PMA; Sigma-Aldrich) solution in dimethyl sulfoxide (DMSO; Sigma-Aldrich) to induce macrophage activation. Luminescence was measured every 2 min for 5 consecutive hours using a Fluoroskan Ascent Microplate Fluorometer (Thermo Fisher Scientific, Breda, The Netherlands) with 1000 ms integration time at 37 °C. The ROS production of infected macrophages was expressed relative to the ROS production of PMA treated macrophages (positive control).

#### Dot blot

Macrophages were seeded, inoculated with *Salmonella* Typhimurium strain 112910a at MOI 100 and incubated in 75 cm² tissue culture treated flasks with filter cap (Greiner Bio-One) as described above for the flowcytometric experiments. At 0 h and 24 h post inoculation, macrophages were scraped off in HBSS + with a cell scraper, brought in a 15 mL tube and centrifuged at 520 × *g* for 10 min at 37 °C. The cells were lysed for 90 min on ice in 1 mL of freshly prepared RIPA buffer composed of 150 mM NaCl (VWR International BVBA, Leuven, Belgium); 1.0% Triton X-100 (Sigma-Aldrich); 0.5% sodium deoxycholate (Sigma-Aldrich); 50 mM Tris pH 8.0 (Sigma-Aldrich); and protease inhibitor cocktail (1:10 diluted; Sigma-Aldrich). Additionally, all samples were sonicated for 4 × 20 s on ice with an Ultrasonic Processor Sonicator XL (Misonix Incorporated, Farmingdale, USA), brought to the same protein concentration using the Bradford-based Protein Assay (Bio-Rad Laboratories, USA), according to the manufacturer’s guidelines, and stored at -80 °C until further use.

Eight μL of each sample was spotted on a Trans-Blot nitrocellulose membrane (Bio-Rad Laboratories) and air dried for 10 min. All subsequent incubation and washing steps were performed on a shaker at room temperature. The membrane was incubated for 30 min in blocking buffer composed of TBS-T buffer (20 mM Tris; 15 mM NaCl; 0.05% Tween-20 (Merck, Schuchardt, Germany) at pH 7.5) with 5% bovine serum albumin (BSA; Sigma-Aldrich). The primary antibody mixture (1:100 diluted mouse anti-pig SLA class II DQ in 0.1% BSA/TBS-T buffer) was applied onto the membrane for 1 h. The membrane was washed 3 × 5 min with TBS-T buffer and subsequently incubated for 1 h with the secondary antibody mixture (1:5000 diluted HRP-conjugated goat anti-mouse IgG (Jackson Immuno Research Laboratories, USA) in 0.1% BSA/TBS-T buffer). After washing the membrane 1 × 15 min and 2 × 5 min in TBS-T and 1 × 5 min in TSB (without Tween-20), the CN/DAB substrate kit (Thermoscientific, USA) was applied onto the membrane, according to the manufacturer’s guidelines. The membrane was scanned with the GS-800 densitometer (Bio-Rad Laboratories) and visually evaluated. In addition, individual spot densities were calculated and expressed as arbitrary densitometry units using the Quantity One software (Bio-Rad Laboratories).

### Level of *hilA*, *sopB*, *ssrA*/*B* and *ssaH* expression in different *Salmonella* strains

The pCS26 plasmid was used for the construction of transcriptional fusions between the promoter regions of *Salmonella* Typhimurium *hilAsopBssrA*/*B* or *ssaH* genes on one hand, and *luxCDABE* genes on the other hand, as decribed before [31,32]. Plasmids with the transcriptional fusions were isolated using the Qiagen Plasmid Midi Kit (Qiagen, Venlo, The Netherlands), electroporated in electrocompetent *Salmonella* bacteria and plated on LB agar with 100 μg/mL kanamycin (Sigma-Aldrich) for selective growth of transformants. Bacteria containing the transcriptional fusions were grown for 16 h in LB with 100 μg/mL kanamycin and 1:10000 diluted in LB. Luminescence was measured every 15 min for 24 h using a Fluoroskan Ascent Microplate Fluorometer (Thermo Fisher Scientific Inc.) with 5000 ms integration time at 37 °C. The extent of *hilAsopBssrA/B* and *ssaH* gene expression is presented as the integral of relative luminescence units (RLU) over a 24 h measurement period.

### Statistical analysis

The capacity to downregulate MHC expression was determined by calculating the average mean FL-5 ratio between *Salmonella* infected and uninfected macrophages at 0 h and 24 h post inoculation (pi) of 3 independent experiments. Differences in MHC expression level between macrophages infected with strain 112910a, either opsonized or not opsonized, and its isogenic mutants at 0 h and 24 h pi were evaluated using a one-way ANOVA on the calculated mean ratios, assuming normal distribution and equal variances. The MHC II expression level of macrophages inoculated with different *Salmonella* strains was evaluated using a paired Student’s *t*-test on the calculated mean ratios at 0 h and 24 h pi. Intracellular proliferation of *Salmonella* strain 112910a in macrophages was evaluated using an independent samples Student’s *t*-test with log CFU per mL as dependent and group as fixed factor.

The invasion capacity of strain 112910a, either opsonized or not with positive or negative pig serum, and its isogenic mutants Δ*hilA* and Δ*hilA*^c^ was compared using a one-way ANOVA, assuming normal distributions and equal variances. ROS production by macrophages infected with *Salmonella* strain 112910a that was either not opsonized, or opsonized with negative or positive pig serum prior to inoculation of macrophages, was analyzed using an unpaired Student’s *t*-test with equal variances not assumed. Differences in *Salmonella* invasiveness and in the effects of *Salmonella* on macrophage viability and ROS production for the different *Salmonella* strains were evaluated using a one-way ANOVA, assuming normal distributions and equal variances. Correlations between MHC expression on one hand, and *Salmonella* invasiveness, macrophage viability, ROS production or *hilA*, *sopB*, *ssrA*/*B* and *ssaH* gene expression on the other hand, were evaluated using the Pearson’s correlation test on the respective datasets. Spot densities from the dot blot assay were evaluated using unpaired two-tailed Student’s *t*-tests and a one-way ANOVA, assuming normal distributions and equal variances.

A *p*-value ≤ 0.05 was considered statistically significant and a value of 0.05 < *p* ≤ 0.10 was considered borderline significant. All statistical analyses were performed using the SPSS 19.0 software (IBM Corporation, New York, USA).

## Results

### *Salmonella* Typhimurium strain 112910a reduces MHC II but not MHC I expression on porcine macrophages in a SPI-1 and SPI-2 dependent way

**Figure 1:**
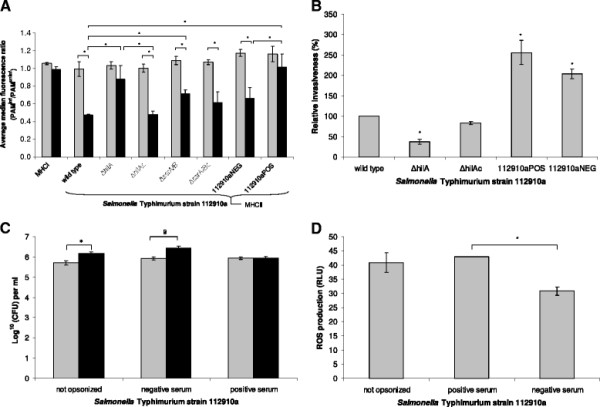
**The capacity of*Salmonella enterica*to downregulate MHC expression on the surface of porcine macrophages,*Salmonella*invasion capacity in macrophages and the effect of opsonization of*Salmonella*with negative and positive pig serum on intracellular proliferation and on reactive oxygen species (ROS) production. A.** The average mean PAM^inf^/PAM^uninf^ ratios (± standard deviation) measured in the FL-5 channel, 0 h (grey bars) and 24 h (black bars) post inoculation (pi) with *Salmonella* Typhimurium strain 112910a or its isogenic deletion mutants Δ*hilA* and Δ*ssrA/B*, and their respective complements Δ*hilA*^c^ and Δ*ssrA/B*^c^. The values were calculated from the ratios obtained from 3 independent experiments and an asterisk refers to a significantly different average mean fluorescence ratio (*p* ≤ 0.05). PAM^inf^ and PAM^uninf^: infected and uninfected porcine alveolar macrophages, respectively. MHC I and MHC II: expression of MHC I or MHC II molecules, respectively, in macrophages inoculated with strain 112910a; 112910aNEG: *Salmonella* Typhimurium strain 112910 opsonized with negative pig serum; 112910aPOS: *Salmonella* Typhimurium strain 112910 opsonized with pig serum containing *Salmonella* Typhimurium specific antibodies. **B.***Salmonella* Typhimurium strain 112910a invasion capacity in porcine macrophages after opsonization with negative and positive pig serum. The relative invasiveness of *Salmonella* Typhimurium *hilA* deletion mutant (Δ*hilA*) and its complement (Δ*hilA*^c^) was used as an internal control. *Salmonella* invasiveness was expressed as the percentage relative to the invasion capacity of strain 112910a ± standard deviations. Each bar represents the average of three independent experiments. An asterisk (*) indicates a significantly different invasion capacity compared to strain 112910a (*p* ≤ 0.05). **C.** The average ^10^log CFU of *Salmonella* Typhimurium strain 112910a that was either not opsonized, opsonized with negative pig serum or opsonized with serum containing *Salmonella* specific antibodies per mL, recovered from macrophages immediately (grey bars) or 5 h (black bars) post inoculation (pi). Bars with an asterisk represent significant bacterial proliferation at 5 h post inoculation (*p* ≤ 0.05). **D.** ROS production of macrophages inoculated with *Salmonella* Typhimurium strain 112910a that was not opsonized or opsonized with negative or positive pig serum. The ROS production of infected macrophages was expressed relative to the ROS production of phorbol 12-myristate 13-acetate (PMA) treated macrophages (positive control). Each bar represents the average of two independent experiments conducted in triplicate. An asterisk (*) indicates a statistically significant difference in ROS production (*p* ≤ 0.05)

To examine the effect of a *Salmonella* Typhimurium strain 112910a infection on MHC I and II expression, the expression of MHC molecules on the surface of 112910a infected macrophages and uninfected macrophages (PAM^112910a^ and PAM^uninf^, respectively) was measured immediately (0 h) and 24 h post inoculation (pi). Ratios were calculated as the average mean ratio of 3 independent experiments. At 0 h, PAM^112910a^/PAM^uninf^ was 1.054 (± 0.011) and 0.990 (± 0.083) for MHC I and MHC II, respectively, corresponding to equal MHC expression levels on uninfected macrophages and macrophages infected with strain 112910a. At 24 h pi, PAM^112910a^/PAM^uninf^ for MHC I did not significantly differ from the average ratio at 0 h pi (*p* > 0.05), suggesting that the MHC I expression level of PAM^112910a^ remained unaffected. In contrast to this, PAM^112910a^/PAM^uninf^ for MHC II expression at 24 h pi was significantly lower than at 0 h (*p* ≤ 0.05), corresponding to a decreased MHC II expression level on PAM^112910a^. These results are summarized in Figure 1A and show that *Salmonella* Typhimurium strain 112910a specifically downregulates MHC II expression on porcine macrophages.

To determine the possible role of *Salmonella* Pathogenicity Island 1 and/or 2 (SPI-1 and SPI-2, respectively) in *Salmonella* induced downregulation of MHC II expression on macrophages, the expression level between uninfected macrophages (PAM^uninf^) and macrophages infected with either strain 112910a (PAM^112910a^), its isogenic deletion mutants Δ*hilA* (PAM^Δ*hilA*^), Δ*ssrA/B* (PAM^Δ*ssrA/B*^) or their respective complements Δ*hilA*^c^ (PAM^Δ*hilAc*^) and Δ*ssrA/B*^c^ (PAM^Δ*ssrA/Bc*^) was compared. At 0 h, mean MHC II expression level ratios between *Salmonella* infected and uninfected macrophages were approximately 1 for macrophages inoculated with strain 112910a or its isogenic mutants and did not significantly differ from each other (*p* > 0.05). At 24 h pi, PAM^Δ*hilA*^/PAM^uninf^ and PAM^Δ*ssrA/B*^/PAM^uninf^ were significantly higher than PAM^112910a^/PAM^uninf^ at the same timepoint (*p* < 0.05), suggesting at least partial inhibition of MHC II downregulation when *hilA* or *ssrA/B* was deleted. Furthermore, complementation of the respective deletion mutants resulted in PAM^Δ*hilA*c^/PAM^uninf^ and PAM^Δ*ssrA/B*c^/PAM^uninf^ 24 h pi that did not significantly differ from PAM^112910a^/PAM^uninf^ at the same timepoint (*p* ≥ 0.05), indicating functional restoration of the wild type phenotype. The fact that PAM^Δ*ssrA/B*^/PAM^uninf^ 24 h pi was still significantly lower than the same ratio at 0 h pi, while this was not the case for PAM^Δ*hilA*^/PAM^uninf^, suggests a more important role for SPI-1 than for SPI-2 in *Salmonella* induced downregulation of MHC II expression on macrophages. These results are summarized in Figure 1A.

### Opsonization of *Salmonella* Typhimurium strain 112910a with *Salmonella* specific antibodies inhibits downregulation of MHC II expression on, limits intracellular proliferation in and enhances ROS production by macrophages

Because *Salmonella* Typhimurium strain 112910a was able to downregulate MHC II expression on porcine macrophages, and might thus interfere with the MHC II dependent pig’s antibody response towards *Salmonella* infection, we wanted to examine if opsonization of *Salmonella* with *Salmonella* specific antibodies was detrimental to the bacterium’s MHC II downregulating and intracellular proliferation capacity in porcine macrophages.

First, we examined the effect of opsonization with serum antibodies on *Salmonella* induced downregulation of MHC II expression on porcine macrophages. Macrophages were inoculated with bacteria opsonized either with pig serum containing anti-*Salmonella* Typhimurium antibodies (PAM^112910aPOS^) or with negative pig serum (PAM^112910aNEG^). At 0 h, average mean PAM^112910a^/PAM^uninf^, PAM^112910aNEG^/PAM^uninf^ and PAM^112910aPOS^/PAM^uninf^ were 0.990 ± 0.043; 1.172 ± 0.088 and 1.161 ± 0.101 and did not significantly differ from each other (*p* > 0.05; Figure 1A). After 24 h incubation, PAM^112910aNEG^/PAM^uninf^ was significantly lower than PAM^112910aNEG^/PAM^uninf^ at 0 h pi on one hand (*p* ≤ 0.05), and than PAM^112910aPOS^/PAM^uninf^ at 24 h pi on the other hand (*p* ≤ 0.05), while PAM^112910aPOS^/PAM^uninf^ at 24 h did not significantly differ from the ratio at 0 h pi (*p* > 0.05). This shows that opsonization of strain 112910a with *Salmonella* specific serum antibodies suppressed the bacterium’s ability to downregulate MHC II expression on macrophages (Figure 1A).

When strain 112910 was opsonized with either positive or negative pig serum prior to infection of macrophages, based on flowcytometrical data, significantly more macrophages were infected compared to macrophages inoculated with non opsonized bacteria (*p* ≤ 0.05; Figure 1B). As an internal control for proper use of flowcytometry to assess the *Salmonella* invasion capacity, the invasion capacity of strain 112910a isogenic mutants Δ*hilA* and Δ*hilA*^c^ was determined. We found that Δ*hilA* was significantly impaired in macrophage invasion compared to the background strain and to its complement Δ*hilA*^c^ (*p* ≤ 0.05; Figure 1B). Using a gentamicin protection assay, it was found that bacteria that were not opsonized or opsonized with negative pig serum were able to significantly proliferate intracellularly 5 h post inoculation of macrophages (*p* ≤ 0.05; Figure 1C). Salmonellae that were opsonized with pig serum containing *Salmonella* specific antibodies, prior to inoculation of macrophages, did not significantly proliferate (*p* > 0.05; Figure 1C). Furthermore, macrophages infected with strain 112910a that was opsonized with positive pig serum showed a tendency towards the production of higher amounts of reactive oxygen species (ROS) than macrophages infected with strain 112910a that was not opsonized or opsonized with negative pig serum prior to inoculation of macrophages (Figure 1D). These differences were only statistically significant between macrophages infected with *Salmonella* opsonized with positive pig serum on one hand, and with *Salmonella* opsonized with negative serum on the other hand (*p* ≤ 0.05).

### Reduction of MHC II expression is *Salmonella* specific and *Salmonella* strain dependent

**Figure 2:**
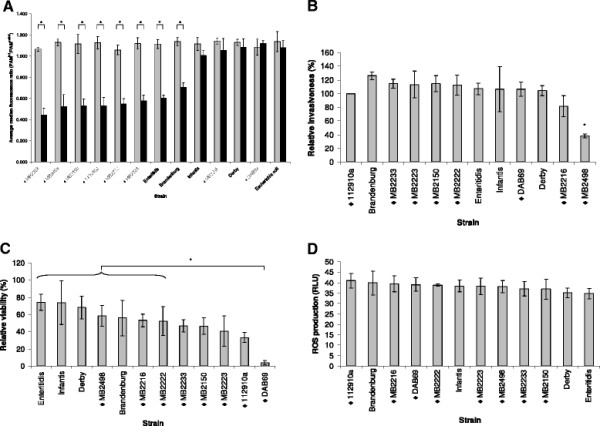
**The capacity of different*Salmonella*strains to downregulate MHC expression on the surface of porcine macrophages, the*Salmonella*invasion capacity in macrophages, and the effect of different*Salmonella*strains on macrophage viability and reactive oxygen species (ROS) production. A.** The average mean PAM^inf^/PAM^uninf^ ratios (± standard deviation) measured in the FL-5 channel, 0 h (grey bars) and 24 h (black bars) post inoculation (pi) with different *Salmonella* strains or an *Escherichia coli* strain. The values were calculated from the ratios obtained from 3 independent experiments and an asterisk refers to a significantly different average mean fluorescence ratio (*p* ≤ 0.05). *Salmonella* Typhimurium strains are indicated with ♦. PAM^inf^ and PAM^uninf^: infected and uninfected porcine alveolar macrophages, respectively. **B.** The invasion capacity in porcine macrophages of different *Salmonella* strains. *Salmonella* Typhimurium strains are indicated with ♦. *Salmonella* invasiveness was expressed as the percentage relative to the invasion capacity of strain 112910a ± standard deviations. Each bar represents the average of three independent experiments. An asterisk (*) indicates a statistically significant difference in invasion capacity (*p* ≤ 0.05). **C.** Macrophage viability is expressed as the average percentage of viable cells at 24 h post inoculation ± standard deviation relative to viable uninfected macrophages (negative control). Each bar represents the average of three independent experiments conducted in triplicate. *Salmonella* Typhimurium strains are indicated with ♦. An asterisk (*) indicates a statistically significant difference in macrophage viability (*p* ≤ 0.05). **D.** ROS production of macrophages inoculated with different *Salmonella* strains. The ROS production of infected macrophages was expressed relative to the ROS production of phorbol 12-myristate 13-acetate (PMA) treated macrophages (positive control). Each bar represents the average of two independent experiments conducted in triplicate. *Salmonella* Typhimurium strains are indicated with ♦

While we found that *Salmonella* Typhimurium strain 112910a was able to downregulate MHC II expression on porcine macrophages, an *Escherichia coli* strain was not (Figure 2A). Because a typical characteristic of *Salmonella* pathogenesis is that it is strongly dependent on host species, serovar and even strain, we verified whether the capacity of *Salmonella* to downregulate MHC II expression on macrophages also differed among *Salmonella* strains. Among the 7 different *Salmonella* Typhimurium pig stool isolates tested, strain MB2216 did not induce MHC II downregulation on macrophages at 24 h pi, while the other strains exhibited downregulation of MHC II expression similar to strain 112910a (Figure 2A). Furthermore, the pigeon isolate *Salmonella* Typhimurium DAB69, the *Salmonella* Derby and *Salmonella* Infantis strains showed no MHC II downregulation on macrophages at 24 h pi, in contrast to the serovar Brandenburg and Enteritidis strains. We can conclude that the capacity of *Salmonella* to downregulate MHC II expression on macrophages is *Salmonella* specific and strain dependent (Figure 2A).

### Strain dependent downregulation of MHC II expression on macrophages does not correlate with differences in *Salmonella* invasion capacity or *Salmonella* induced cytotoxicity and reactive oxygen species (ROS) production

We verified whether the observed strain dependent capacity to downregulate MHC II expression on porcine macrophages correlated with *Salmonella* invasion capacity or altered macrophage viability or activation status.

**Figure 3:**
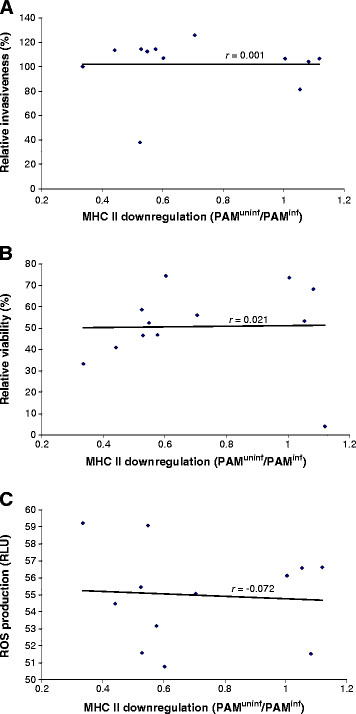
**Correlation between MHC II downregulation and invasion capacity, cytotoxicity or ROS production.** Correlation between *Salmonella* induced MHC II downregulation (X-axis) and (**A**) *Salmonella* invasion capacity, (**B**) macrophage viability or (**C**) ROS production (on the respective Y-axes) with *r* = Pearson correlation coefficient. MHC II downregulation capacity is presented as the average mean FL-5 signal ratios between infected and uninfected porcine alveolar macrophages (PAM^inf^ and PAM^uninf^, respectively). *Salmonella* invasion capacity is expressed as the number of macrophages infected by a particular *Salmonella* strain relative to the macrophages infected by strain 112910a. Viability is expressed as the average percentage of viable cells at 24 h post inoculation relative to viable uninfected macrophages (negative control). ROS production of infected macrophages was expressed relative to the ROS production of phorbol 12-myristate 13-acetate (PMA) treated macrophages (positive control). ROS = reactive oxygen species; PAM = porcine alveolar macrophages

First, we compared the invasion capacity of all tested *Salmonella* strains. *Salmonella* Typhimurium strain MB2498 was significantly less able to infect macrophages compared to the other *Salmonella* strains (*p* ≤ 0.05; Figure 2B), but was able to downregulate MHC II expression comparably to, or even better than strains that showed higher infection rates (Figure 2A). We did not find a significant correlation between invasion capacity and the extent of downregulation of MHC II expression (*r* = 0.001; Figure 3A). Furthermore, we found that viability of macrophages inoculated with the tested strains differed, and that viability of DAB69 inoculated macrophages was dramatically decreased 24 h post inoculation (Figure 2C). However, no significant correlation was found between MHC II expression level and cell viability at 24 h (*r* = 0.021; Figure 3B). Finally, infection of macrophages with the different *Salmonella* strains did not significantly affect ROS production (*p* > 0.05; Figure 2D) and no significant correlation was detected between MHC II downregulation capacity and ROS production (*r* = -0.072; Figure 3C).

### Differential expression of SPI-1 and SPI-2 genes does not correlate with strain dependent downregulation of MHC II expression

**Figure 4:**
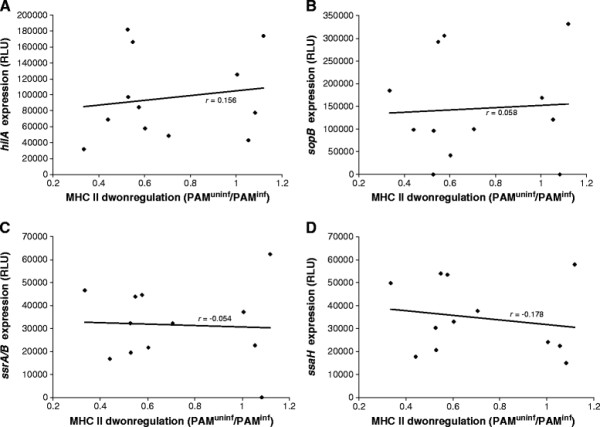
**Correlation between MHC II downregulation and SPI-1 or SPI-2 gene expression.** Correlation between MHC II downregulation capacity (X-axis) and expression of *Salmonella* Typhimurium SPI-1 genes *hilA* (**A**) and *sopB* (**B**) and SPI-2 genes *ssrA/B* (**C**) and *ssaH* (**D**) (on the respective Y-axes) by different *Salmonella* strains, with *r* = Pearson correlation coefficient. Gene expression was measured as the luminescence of the respective *Salmonella* strain transformed with a plasmid in which the *hilA*, *sopB*, *ssrA*/*B* or *ssaH* promoter was cloned upstream of the reporter genes *luxCDABE*. Luminescence (and expression of the virulence genes) was measured in real time with a Fluoroskan luminometer during 24 h incubation of these strains at 37 °C in LB medium. MHC II downregulation capacity is presented as the average mean FL-5 signal ratios between infected and uninfected porcine alveolar macrophages (PAM^inf^ and PAM^uninf^, respectively). Gene expression is presented as the average relative luminescence units (RLU) of three independent measurements conducted in triplicate ± standard deviation

We showed that *Salmonella* Typhimurium strain 112910a induced downregulation of MHC II expression was at least partially dependent on SPI-1 and SPI-2 gene expression. Therefore we wanted to examine if the strain dependent differences in the capacity to downregulate MHC II expression on macrophages correlated with an altered expression of SPI-1 and/or SPI-2 genes. We found that various *Salmonella* strains expressed SPI-1 genes *hilA* and *sopB* to a different extent. Expression of *sopB* could not be detected in the *Salmonella* Typhimurium MB2498 and *Salmonella* Derby strains. No significant correlation was found between the expression levels of both genes in the different *Salmonella* strains [unpublished observations]. Expression of SPI-2 genes *ssrA/B* and *ssaH* also differed between *Salmonella* strains, but in contrast to expression of both SPI-1 genes, a statistically significant positive correlation was found between *ssrA/B* and *ssaH* expression in the strains (*p* < 0.05 with *r* = 0.89) [unpublished observations]. However, no significant correlation was found between *hilA*, *sopB*, *ssrA/B* or *ssaH* gene expression and the capacity to downregulate MHC II expression for the tested *Salmonella* strains (Figure 4A-D).

### Dot blot

**Figure 5:**
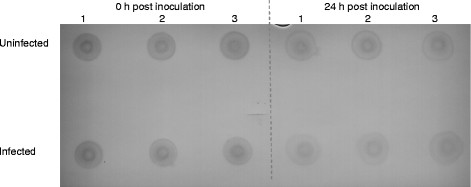
**The total amount of MHC II molecules in porcine macrophages is decreased 24 h post inoculation with*Salmonella*Typhimurium strain 112910a.** Macrophages were either or not inoculated with *Salmonella* Typhimurium strain 112910a at MOI 10. Whole cell lysates were prepared at 0 and 24 h post inoculation. Eight μL of each lysate were spotted on a nitrocellulose membrane and air dried. The membrane was incubated with a primary mouse anti-pig SLA class II DQ antibody, and subsequently with a secondary HRP-conjugated goat anti-mouse IgG antibody. The CN/DAB substrate kit was applied onto the membrane and the membrane was scanned with a GS-800 densitometer

Based on visual analysis of the dot blot, the total amount of MHC II is less 24 h post inoculation in both uninfected and infected macrophages compared to 0 h pi, with less MHC II molecules in *Salmonella* infected macrophages 24 h pi compared to uninfected macrophages 24 h pi (Figure 5). In addition, spot densities were calculated using the Quantity One software, however, no statistically significant differences between the groups were detected [unpublished observations]. We therefore conclude that the overall amount of MHC II molecules is not significantly decreased 24 h post inoculation of macrophages.

## Discussion

At present, preharvest *Salmonella* monitoring programs are primarily based on serological analysis, an inexpensive method that allows rapid screening of high numbers of pig herds [6]. The onset of seroconversion after *Salmonella* infection in pigs is difficult to predict and can range from 6 days up to 25 weeks after oral inoculation, depending on, among others, the inoculation strain and inoculation dose used and piglet age [7,9,33,34]. Yet, no data are available about the underlying mechanisms that account for these differences.

Seroconversion is initiated when antigen-presenting cells like macrophages and dendritic cells present pathogen-derived peptide antigens to T cells in 2 different ways. In the endogenous pathway, polypeptides that access the host cell’s cytosol are degraded by the proteasome and are presented at the surface of antigen presenting cells through MHC I molecules. *Salmonella*, however, enters the host cell through the endocytic route, causing its peptides to be presented via MHC II molecules after lysosomal degradation [35,36]. Antigens bound to MHC II are presented to naïve CD4+ helper T cells that will develop into effector T cells. The latter than differentiate into two major subtypes of cells known as type 1 and type 2 helper T cells (T_h_1 and T_h_2 cells, respectively), depending on, among others, the chemo- and cytokine environment. T_h_1 cells predominantly activate macrophages while T_h_2 cells will induce maturation of B cells to antibody producing plasma cells. Because the MHC II antigen presenting pathway is able to mount both a cellular and/or humoral immune response, bacteria that interfere with the MHC II expression might radically influence the host’s immune response towards infection. Generally, the expression of MHC II molecules on the surface of macrophages is upregulated following ingestion of microorganisms and recognition of their foreign molecular pattern [37]. We found that *Salmonella* Typhimurium strain 112910a specifically downregulated MHC II expression on the surface of macrophages and that the MHC I level of macrophages remained unaffected 24 h post inoculation (pi). These results suggest that *Salmonella* specifically interferes with the MHC II presentation pathway in porcine macrophages, leaving the MHC I pathway undisturbed. Furthermore, downregulation of MHC II expression on porcine macrophages turned out to be *Salmonella* specific, since we found that an *Escherichia coli* strain, another closely to *Salmonella* related member of the Enterobacteriaceae family, was unable to induce MHC II downregulation. Inoculation of bovine macrophages with *Salmonella* Typhimurium did not cause downregulation of MHC II expression [38]. Although porcine alveolar macrophages represent a quite specific model which might explain their differential behaviour compared to macrophages or other cell types from other host species, our findings might provide further evidence for host-specific *Salmonella* behaviour.

*Salmonella* Pathogenicity Islands (SPIs’) are clusters of genes encoding virulence factors that play an important role at different stages of the pathogenesis of *Salmonella* infections in pigs [5]. We examined whether SPI-1 and/or SPI-2 were involved in the observed *Salmonella* induced downregulation of MHC II expression on macrophages. *Salmonella* Typhimurium SPI-1 plays a crucial role in the colonization and invasion of the porcine gut [39], while SPI-2 is predominantly involved in replication and survival of *Salmonella* Typhimurium in porcine macrophages [20]. HilA and SsrA/B are major regulators of SPI-1 and SPI-2, respectively, and both regulators induce transcriptional activation of downstream genes in response to a variety of stimuli [23,40]. We found that *Salmonella* Typhimurium induced downregulation of MHC II expression was at least partly dependent on SPI-1 and SPI-2. Since recent evidence shows that SPI-1 is also involved in intracellular behaviour of *Salmonella* in macrophages, besides its main role in cell invasion [36], the fact that MHC II downregulation is inhibited when SPI-1 is abolished is not a complete surprise. Both SPI-1 and SPI-2 encode a type 3 secretion system, T3SS-1 and T3SS-2 respectively, a needle-like structure that is used to inject so called ‘effector proteins’ into the host cell’s cytosol, for uptake of the bacterium or to adapt the intracellular environment in function of *Salmonella* survival. Mitchell and colleagues already showed that SPI-2 effector SifA plays a role in downregulation of MHC II expression on human Mel JuSo cells, possibly by recruiting vesicles that transport MHC II molecules to the cell membrane, to maintain the integrity of the *Salmonella* containing vacuole (SCV) [12]. In support of the hypothesis of redistribution of MHC II molecules from the cell surface to the SCV, our data from the dot blot assay suggest no change in the total amount of MHC II molecules in uninfected and *Salmonella* infected macrophages 24 h post inoculation. It is likely that SPI-1 and SPI-2 secreted effector proteins cause the *Salmonella* induced downregulation of MHC II expression on porcine macrophages. Altogether, we showed an explicit role for SPI-1 and a more discreet role for SPI-2 in downregulation of MHC II expression on porcine macrophages. However, because MHC II expression levels in Δ*hilA* and Δ*ssrA/B* infected macrophages were not completely restored, other factors than SPI-1 and SPI-2 might be involved as well.

It has commonly been assumed that resistance to facultatively intracellular bacteria like *Salmonella* Typhimurium predominantly requires a cellular immune response. However, there appear to be multiple mechanisms by which antibodies can influence the course of infection with such pathogens [41]. We found that, when *Salmonella* Typhimurium strain 112910a was opsonized with *Salmonella* specific antibodies prior to inoculation of macrophages, the bacterium lost its capacity to interfere with MHC II expression and was less able to proliferate in porcine macrophages. A proposed mechanism by which antibodies might interfere with *Salmonella* virulence is by sterically hindering the proper insertion of T3SS-1 and/or T3SS-2, since both secretion systems are required for successful intracellular proliferation of *Salmonella*[36].

*Salmonella* can actively invade macrophages using T3SS-1 or can be taken up passively through Fc-receptor mediated phagocytosis [42]. Besides an effect through sterical hindrance of T3SS, it is thus possible that opsonization of *Salmonella* with serum antibodies favoured phagocytosis over *Salmonella* controlled invasion. It was found that when *Salmonella* was opsonized prior to inoculation of macrophages, approximately 2 to 2.5 times more bacteria were internalized than in macrophages that were inoculated with non opsonized *Salmonella*. Salmonellae internalized into macrophages reside within *Salmonella* containing vacuoles (SCV) and the environment in these SCV after Fc-receptor mediated phagocytosis is different from that after T3SS-1 dependent invasion [42]. These different environmental changes might subsequently result in differences in the expression of *hilA* and other *Salmonella* genes, and in this way alter the intracellular behaviour of the microorganism [32,43,44]. Since we showed that downregulation of MHC II expression is SPI-1 and SPI-2 dependent, differential gene expression caused by the used internalization mechanism might affect the extent of *Salmonella* interference with the MHC II expression on macrophages. The mechanism of *Salmonella* internalization might furthermore affect the macrophage activation level. Indeed, macrophages that phagocytosed *Salmonella* that was opsonized with *Salmonella* specific antibodies produced more ROS than macrophages infected with bacteria that were not opsonized, or opsonized with negative pig serum, indicating increased macrophage activation [45]. Furthermore, enhanced ROS production can result in growth inhibition of intracellular bacteria and even destruction of these bacteria [20], which might explain why *Salmonella* was less able to proliferate intracellularly after opsonization with *Salmonella* specific antibodies. Inhibition of downregulation of MHC II expression when *Salmonella* was opsonized with antibodies prior to inoculation of macrophages might, in addition to sterically hindering the proper insertion of T3SS-1 and/or T3SS-2, thus be the result of enhanced phagocytosis and/or ROS production and subsequent changes in *Salmonella* virulence gene expression. The findings that opsonization of *Salmonella* with specific antibodies prior to inoculation of porcine macrophages limits intracellular proliferation and inhibits downregulation of MHC II expression emphasize the importance for *Salmonella* to circumvent antibody production for successful persistence in the pig host.

Since strain and host specific behaviour is a key characteristic of *Salmonella* pathogenesis [46,47], we determined if other *Salmonella* isolates exhibited the same phenotype as strain 112910a. We found that the extent of MHC II downregulation differed considerably among various *Salmonella* strains. *Salmonella* Typhimurium strain MB2216, a pig isolate, and strain DAB69, a pigeon isolate, and serovar Infantis and Derby strains, both pig isolates, exhibited no downregulation of MHC II expression in the current setup.

Subsequently, it was determined if the strain specific differences in MHC II downregulation capacity were inherent to macrophage infection by that particular strain, or correlated with *Salmonella* invasion capacity in, *Salmonella* induced cytotoxicity on and ROS production by macrophages. The *Salmonella* invasion capacity of the tested *Salmonella* strains did not significantly differ, except for strain MB2498 that was significantly impaired in infecting macrophages. This might be due to the fact that this strain was markedly less motile than the other strains, as assessed by growing the strains in semi solid agar [48,49]. Infection with all tested *Salmonella* strains was cytotoxic for macrophages 24 h pi. Finally, the macrophage activation status indicated by ROS production measurement did not differ between *Salmonella* strains. Because we found no significant correlation between the extent of MHC II downregulation on one hand, and *Salmonella* invasiveness, cytotoxicity or induction of ROS production on the other hand, we can conclude that strain specific differences in the capacity to downregulate MHC II expression on porcine macrophages are irrespective of strain specific differences in invasion capacity and cytotoxic effects on or induction of ROS production by macrophages.

Since we showed that SPI-1 and SPI-2 are involved in *Salmonella* Typhimurium strain 112910a induced downregulation of MHC II expression, we wanted to examine if differential SPI-1 and/or SPI-2 gene expression accounted for the observed differences in MHC II downregulation between *Salmonella* strains. Surprisingly, no correlation was found between the expression of SPI-1 genes *hilA* and *sopB* for a certain strain, although this can be due to the fact that expression of the effector protein SopB is only partly regulated by HilA [22]. Furthermore, no *sopB* expression was detected in *Salmonella* MB2498 and Derby strains, indicating the absence of the *sopB* containing SPI-5 in these strains [13,50]. Expression of SPI-2 genes *ssrA/B* and *ssaH* markedly differed between *Salmonella* strains, and in contrast to *hilA* and *sopB* expression, a significantly positive correlation was found between *ssrA/B* and *ssaH* expression in a certain strain. However, no correlation was found between SPI-1 or SPI-2 gene expression and the capacity to downregulate MHC II expression. Although *Salmonella* gene expression in LB medium and inside macrophages might vary, the fact that SPI-1 and SPI-2 gene expression patterns of different *Salmonella* strains did not correlate with their capacity to interfere with the MHC II expression level of macrophages might indicate that other factors than SPI-1 and SPI-2 are also involved in *Salmonella* induced MHC II downregulation.

In conclusion, we showed that *Salmonella* Typhimurium strain 112910a specifically downregulated MHC II expression on porcine macrophages in merely a SPI-1 and SPI-2 dependent way. Furthermore, opsonization of *Salmonella* with antibodies counterbalanced the bacterium’s capacity to downregulate MHC II expression and to proliferate intracellularly, emphasizing the importance for *Salmonella* to interfere with the host’s immune response for successful persistence. The extent of MHC II downregulation on macrophages differed among *Salmonella* strains and these strain specific differences did not correlate with differential SPI-1 and/or SPI-2 expression, providing evidence that besides SPI-1 and SPI-2 other factors are also involved in *Salmonella* induced downregulation of MHC II expression. Our findings might imply that *Salmonella* strains that are capable of circumventing the pig’s immune response can better persist in pigs, interfering with serological screenings that are used in most preharvest *Salmonella* monitoring programs in Europe to date.

## Competing interests

Sources of financial support have been acknowledged and the authors declare that they have no competing interests.

## Authors’ contributions

AVP, FB, FP and FH participated in the design of the study. AVP, BF, EV and BL performed the experiments and AVP and BF analysed the data. AVP, FP, FB and FH wrote the manuscript. All authors read and approved the final manuscript.
